# Contrasted phylogeographic patterns of hydrothermal vent gastropods along South West Pacific: Woodlark Basin, a possible contact zone and/or stepping-stone

**DOI:** 10.1371/journal.pone.0275638

**Published:** 2022-10-05

**Authors:** Camille Poitrimol, Éric Thiébaut, Claire Daguin-Thiébaut, Anne-Sophie Le Port, Marion Ballenghien, Adrien Tran Lu Y, Didier Jollivet, Stéphane Hourdez, Marjolaine Matabos

**Affiliations:** 1 Adaptation et Diversité en Milieu Marin, Station Biologique de Roscoff, Sorbonne Université, CNRS, Roscoff, France; 2 Biologie et Ecologie des Ecosystèmes marins Profonds, Ifremer, CNRS, UBO, Plouzané, France; 3 Institut des Sciences de l’Evolution de Montpellier, Université Montpellier, CNRS, EPHE, IRD, Montpellier, France; 4 Laboratoire d’Ecogéochimie des Environnements Benthiques, Observatoire Océanologique de Banyuls, Sorbonne Université, CNRS, Banyuls-sur-Mer, France; Academia Sinica, TAIWAN

## Abstract

Understanding drivers of biodiversity patterns is essential to evaluate the potential impact of deep-sea mining on ecosystems resilience. While the South West Pacific forms an independent biogeographic province for hydrothermal vent fauna, different degrees of connectivity among basins were previously reported for a variety of species depending on their ability to disperse. In this study, we compared phylogeographic patterns of several vent gastropods across South West Pacific back-arc basins and the newly-discovered La Scala site on the Woodlark Ridge by analysing their genetic divergence using a barcoding approach. We focused on six genera of vent gastropods widely distributed in the region: *Lepetodrilus*, *Symmetromphalus*, *Lamellomphalus*, *Shinkailepas*, *Desbruyeresia* and *Provanna*. A wide-range sampling was conducted at different vent fields across the Futuna Volcanic Arc, the Manus, Woodlark, North Fiji, and Lau Basins, during the CHUBACARC cruise in 2019. The *Cox1*-based genetic structure of geographic populations was examined for each taxon to delineate putative cryptic species and assess potential barriers or contact zones between basins. Results showed contrasted phylogeographic patterns among species, even between closely related species. While some species are widely distributed across basins (i.e. *Shinkailepas tollmanni*, *Desbruyeresia melanioides* and *Lamellomphalus*) without evidence of strong barriers to gene flow, others are restricted to one (i.e. *Shinkailepas tufari* complex of cryptic species, *Desbruyeresia cancellata* and *D*. *costata*). Other species showed intermediate patterns of isolation with different lineages separating the Manus Basin from the Lau/North Fiji Basins (i.e. *Lepetodrilus schrolli*, *Provanna and Symmetromphalus* spp.). Individuals from the Woodlark Basin were either endemic to this area (though possibly representing intermediate OTUs between the Manus Basin and the other eastern basins populations) or, coming into contact from these basins, highlighting the stepping-stone role of the Woodlark Basin in the dispersal of the South West Pacific vent fauna. Results are discussed according to the dispersal ability of species and the geological history of the South West Pacific.

## Introduction

Among deep-sea environments, hydrothermal vents are found in different geological contexts including mid-oceanic ridges, back-arc basins, volcanic arcs, and active seamounts [1]. Mineral deposits described in these environments contain high concentrations of metals that are of increasing interest to mining companies [2]. Although not yet started, mining activities are expected to strongly impact vent communities which harbour highly specialized endemic fauna that relies on local microbial chemosynthesis [3]. The potential impacts are either direct or indirect, and include habitat destruction and complete removal of organisms living there, but also the creation of a sediment plume that will affect several chemical and biological processes for the underlying fauna [4–6]. In the South West Pacific, the mineral richness of vent sites and their close proximity to the coastline led to a large number of exploration and exploitation mining leases in the region [7, 8]. Because ecosystem resilience strongly depends on the vent fauna capacity to recolonize sites, informing future exploitation of massive sulphide mounds requires a good understanding of species distribution and how they are connected among sites [9]. Examining biogeographic patterns of the vent fauna at the scale of the western Pacific is thus essential to describe current species ranges.

Current biogeographical patterns reflect the constant reorganisation of ridges, changes in ocean floor geomorphology and opening/closing of basins, some of which erased by subduction processes, making their identification difficult [10, 11]. To date, eleven biogeographic provinces have been reported for the hydrothermal vent fauna based on taxon composition [7], but their delineation faces several issues. First, these provinces are based on an incomplete inventory of the diversity of the hydrothermal vent fauna and species spatial distribution. Secondly, in addition to the decreasing number of taxonomists, it can be difficult for ecologists to identify with confidence vent species based on taxonomic descriptions that do not always report on morphological ontogenic changes and, in the case of mollusks, are often based on shell morphology of only a few specimens. In mollusks, the strong morphological plasticity of the shell (e.g as reported by Chen et al. [12]) and the morphological stasis of species can lead to species misidentification [13–15] and consequently the overestimation of the geographic range of species [16]. In addition, an increasing number of DNA-barcoding studies have highlighted the presence of well-separated cryptic vent species among ridge segments due to geographical barriers [14, 17–20]. Finally, heterogeneity in sampling efforts also leads to a lack of information on key zones to properly assess species range [21].

The South West Pacific region which forms an independent biogeographic province comprises relatively recent back-arc basins and volcanic arcs (i.e. < 10 million years old) that present various geological and geodynamic contexts [22–24]. Although data on larval distribution and indirect estimates of larval dispersal provided by biophysical models of water masses circulation and phylogeographic analyses have contributed to a better understanding of population connectivity along mid-oceanic ridges [25–27], less is known for the back-arc basin discontinuous system. The complex geological history of these basins and present-day hydrodynamic regimes may limit larval dispersal within and between basins [28]. A modelling study reported various patterns of connectivity depending on the planktonic larval duration and the dispersal depth with: (1) a lack of connectivity between the Manus Basin and those of North Fiji and Lau with the Woodlark Basin acting possibly as a stepping-stone, and (2) a strong connection of nearby basins [28]. At intermediate spatial scales, this study suggested unidirectional larval transport from the Woodlark Basin to the Manus Basin and from the Lau Basin to the North Fiji Basin. The Solomon and New Hebrides Basins, two newly-opened back-arc basins located between the Woodlark Basin and the North Fiji Basin, may also represent possible stepping-stones to connect eastern and western basins. Conversely, vent fields are often well connected within a basin with no directionality.

Genetic studies on several gastropods and crustaceans based on cytochrome oxidase I gene (*Cox1*) and/or microsatellite loci confirmed the occurrence of variable phylogeographic patterns from large scale connectivity with only one panmictic population [29] to strong regional genetic differences that separates the Manus Basin populations from those of the North Fiji and Lau Basins [30–32]. Contrasted geographic ranges have also been observed for closely related species. For example, amongst the three different species of the snail *Alviniconcha* found in the South West Pacific, *A*. *kojimai* and *A*. *boucheti* are largely distributed over the Manus, North Fiji, and Lau Basins, and the Futuna Volcanic Arc, while *A*. *strummeri* distribution is limited to the Futuna Arc and the Lau Basin [13, 33, 34]. The *Lepetodrilus schrolli* species complex is another example of taxa composed of cryptic species in the region [17], with *Lepetodrilus schrolli* only found in the Manus Basin while *Lepetodrilus* aff. *schrolli* is present in both the North Fiji/Lau and Manus Basins, but with a strong genetic differentiation between its eastern (North Fiji/Lau Basins) and western (Manus Basin) populations [19]. These results question the link between the Manus Basin and the more eastern basins including the potential barriers to dispersal or the presence of intermediate stepping-stone sites.

In the South West Pacific, most studies on the diversity of vent ecosystems have focused on a few basins, i.e. the Manus, Lau and North Fiji Basins while some other active areas are insufficiently known, in particular the Woodlark Basin, or the Solomon and Vanuatu (ex New Hebrides) Trench [28]. Very recently, hydrothermal vent communities have been discovered and described for the first time in the Woodlark Basin [35]. These communities are not profoundly different from communities reported in other western Pacific back-arc basins. Preliminary barcoding analyses of the main engineer species suggested that the Woodlark Basin may act as a biodiversity dispersion centre for the hydrothermal vent fauna and a crossroad between the East/West basins in this Pacific region. In this context, the aims of our study were to: (1) perform a barcoding analysis to confirm morphological descriptions of gastropod species and identify potential cryptic species; (2) compare the phylogeographic patterns of a range of families at the regional scale of the South West Pacific. The final aim of the study was therefore to contribute to a better understanding of species distribution and connectivity across the Manus, Woodlark, North Fiji, and Lau Basins, and the Futuna Volcanic Arc. We focused on six genera from four families of vent gastropods widely distributed in the region: the Lepetodrilidae *Lepetodrilus*, the Neomphaloidae *Symmetromphalus* and *Lamellomphallus*, the Phenacolepadidae *Shinkailepas*, and the Provannidae *Desbruyeresia* and *Provanna*.

## Materials and methods

### Ethics statement

Gastropods used in this study were collected with necessary authority permissions of the foreign countries. Permission for sampling in Exclusive Economic Zones (EEZ) was issued by the Papua New Guinea, The Republic of Fiji and Kingdom of Tonga. We obtained the agreement to sample in Wallis et Futuna waters by the Haut Commissariat à la République in New Caledonia and the Préfecture in Wallis and Futuna. We also made an official request under the Nagoya agreements for the use of samples for genetic academic researches.

### Sampling

**Fig 1:**
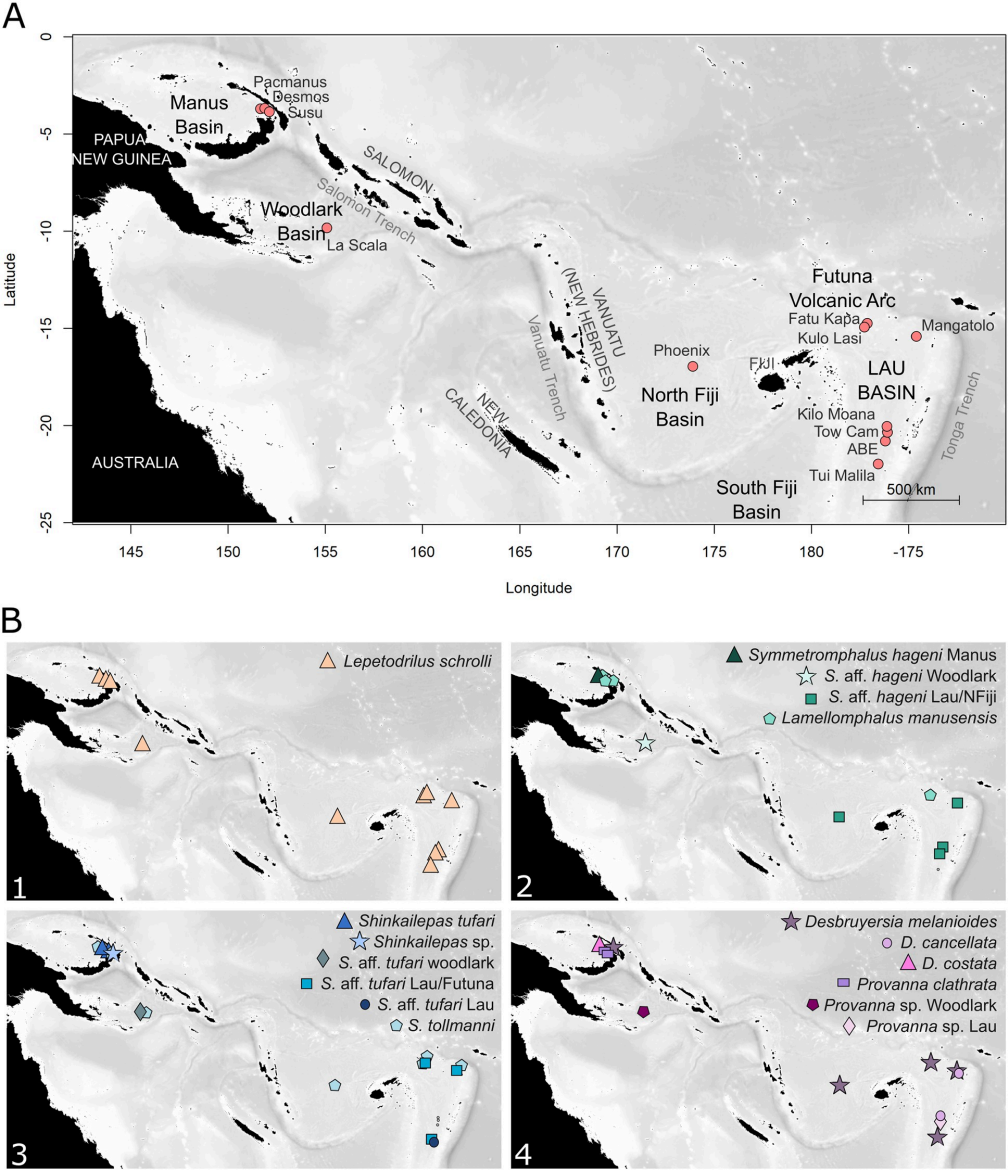
Back-arc-basins sampling area in the South West Pacific (A) and sampling location of the various gastropod species from the Lepetodrilidae (B1), Neomphaloidae (B2), Phenacolepadidae (B3) and Provannidae (B4) families. Red dots represent sampled fields.

A wide-range sampling of vent species was conducted during the CHUBACARC cruise held between March and June 2019 in the southwestern Pacific back-arc basins aboard the French research vessel *L’Atalante* [36]. Vent fauna was collected in four distinct habitats defined based on engineer species along a gradient of venting (i.e. *Bathymodiolus* beds, *Ifremeria* aggregations, *Alviniconcha* aggregations, *Arcovestia* clumps), and representative of the sampled sites. Collections were made using the hydraulic arm and the suction sampler of the ROV *Victor 6000* and transferred in either insulated bioboxes or the suction sampler device containers. One to five vent fields were sampled within each basin, representing a total of twelve vent fields in the Lau, North Fiji, Manus and Woodlark Basins, and the Futuna Volcanic Arc (Fig 1A and 1B, Table 1). Samples were washed through a 250 μm sieve and gastropods from six genera (i.e. *Lepetodrilus*, S*ymmetromphalus*, *Lamellomphalus*, *Shinkailepas*, *Desbruyeresia* and *Provanna*) sorted on board and stored in 96% ethanol until DNA extraction. Individuals were then morphologically identified to the lowest possible taxonomic level using species taxonomic morphological descriptions [37–43]. Additional specimens from the Kermadec Volcanic Arc were included for some groups. These specimens were collected during the Hydrothermadec cruise (R/V Sonne, chief scientist Andre Kochinsky).

**Table 1:** Location of gastropods sampled along the South West Pacific back-arc basins and number of individuals used for DNA barcoding.

Basin	Vent field	Coordinates	Number of individuals						
			*Lepetodrilus schrolli complex*	*Symmetromphalus hageni complex*	*Lamellomphalus manusensis*	*Shinkailepas tufari* complex	*Shinkailepas tollmanni*	*Desbruyeresia spp*.	*Provanna spp*.
**Manus**	Pacmanus	03° 43’ S 151° 40’ E	6	9	5	4	15	5	-
	Desmos	03° 41’ S 151° 51’ E	16	-	3	-	-	-	5
	Susu	03° 48’ S 152° 06’ E	10	-	16	17	11	1	5
**Woodlark**	La Scala	09° 48’ S 155° 03’ E	22	8	-	9	8	-	8
**North Fiji**	Phoenix	16°57’ S 173° 55’ E	4	8	-	-	8	5	-
**Futuna**	Fatu kapa	14° 45’ S 177°10’ W	4	-	-	1	10	1	-
	Kulo Lasi	14° 56’ S 177° 15’ W	26	-	5	-	9	-	-
**Lau**	Mangatolo	15° 24’ S 174° 39’ W	3	1	-	1	14	5	-
	Kilo Moana	20° 03’ S 176° 08’ W	-	-	-	-	-	3	-
	Tow Cam	20° 19’ S 176° 08’ W	8	4	-	-	-	4	7
	ABE	20° 45’ S 176° 11’ W	3	3	-	-	-	-	-
	Tui Malila	21° 59’ S 176° 24’ W	5	-	-	8	-	-	-
**Kermadec** *	Brothers Caldera	34° 51’ S 179° 03’ E	4	-	3	-	-	-	-

*Samples collected from Hydrothermadec cruise

### Molecular methods

For each species, a subset of specimens was selected from each location where they were present (Fig 1B, Table 1). Except for *Shinkailepas tollmanni* and *Lepetodrilus schrolli* and *L*. aff. *schrolli* individuals for which DNA extraction was performed on board just after sampling (and for which the shell was further stored in ethanol after tissue lysis for further identification and possible shell biometry), a picture of the full specimen (shell and soft tissues) was taken for each individual using a DMC 4500 camera mounted on a LEICA M165 C stereoscopic microscope. DNA was extracted from the foot or the whole body depending on the individual size. If possible, the head and radula were saved to refine species identification based on morphology. For *S*. *tufari*, *Provanna* spp., *Desbruyeresia* spp., *Symmetromphalus* sp., and *Lamellomphalus*, DNA was extracted using NucleoSpin^®^ 96 Tissue kit according to the manufacturer’s instructions, and eluted in 100 μl of elution buffer. A CTAB/PVP extraction procedure (derived from Doyle and Doyle [44]: see Jolly et al. [45]) was used for *S*. *tollmanni* and *Lepetodrilus* sp., and DNA pellets were resuspended in 100 μl of Tris-HCl 5 mM pH 8 buffer.

For each taxon, a fragment of the *Cox1* mitochondrial gene was amplified using the universally-applicable primers LCO1490 and HCO2198 [46]. In case these primers did not yield a clear amplification signal, degenerate versions of these primers, which accounted for the high level of polymorphism often found in marine mollusks (C. Daguin-Thiébaut, unpublished) were also used (Table 2). DNA amplification was performed with a T100 thermocycler (Biorad) by using a 25 μl reaction volume containing DNA, 1X GoTaq^®^ Flexi Buffer (Promega), 0.05 mg/μl of Bovine Serum Albumin, 2 mM MgCl_2_, 0.12 mM of each dNTP, 600 nM of each primer, 0.5 U of GoTaq^®^ G2 Flexi DNA polymerase (Promega) and ultrapure water. Polymerase Chain Reactions were performed as follows: (1) a 3 min initial denaturation at 94°C; followed by (2) 36 cycles including a 30 s denaturation at 94°C, a 30 s primer annealing at 46°C and a 1 min elongation at 72°C; and (3) a final 10 min elongation at 72°C.

**Table 2:** Primers used in the present study for each taxon.

Taxon	Primers	Primer sequences (5’ - 3’)	References
*S*. *tufari* complex	LCO1490	GGTCAACAAATCATAAAGATATTGG	[46]
*S*. *tollmanni*			
*Symmetromphalus*	HCO2198	TAAACTTCAGGGTGACCAAAAAATCA	
*Lamellomphalus*			
*Desbruyeresia*	LCO1490bathy*	GTTCTACAAAYCATAAAGAYATTGG	This study
*Provanna*	HCO2198bathy*	TAAACYTCTGGATGMCCRAARAAYCA	This study
*Lepetodrilus*	LCO1490lepetofam	TTTCMACTAAYCATAAAGACATYGG	This study
	LCO2198schrolnux	TANACTTCTGGRTGRCCRAARAATCA	This study

* in addition to the Folmer original primers, for some samples. See information within Genbank submitted sequences.

The PCR products were then sent to Eurofins Genomics (Ebersberg, Germany) for bidirectional Sanger sequencing. Raw sequence electrophoregrams were carefully checked with Codon Code Aligner v5.1.5 before forward and reverse sequences were assembled, aligned, edited and trimmed using BioEdit v7.2.5 [47].

All sequences with geographic details are available in the GenBank database with accession numbers OK635351-374, OK635388-416, OK635475-499, OK635506-573, OK576792-867, OM264371-375, OM791835-837, OM865896-6006 (see also Table 3).

**Table 3:** List of accession numbers from gastropod individuals used for phylogenetic trees.

Genus	GenBank Accession numbers	References
*Lepetodrilus*	OM865896-6006	This study
	EU306431-444, EU306451-456	[17]
	MZ509428-430, MW497312-314, MW807763-765	[48]
*Symmetromphalus*	OK635541-573	This study
	MW497416	[48]
*Lamellomphalus*	OK635388-416	This study
	OM791835-837	This study
	KY399885	[43]
	AB330999	[49]
*Shinkailepas*	OK635506-540, OM264371-375	This study
	OK576792-867	This study
	LC549687-LC549719	[29]
	LC215328-331, LC387564	[50]
	LC215293-295	[51]
	LC178463-464	[52]
	MW807774-777, MW497415	[48]
*Desbruyeresia*	OK635351-374	This study
	GQ290596	[53]
	MW497309-311	[48]
	MK560876	[40]
	LC090201-203	[54]
*Provanna*	OK635475-499	This study
	LC095875	[55]
	GQ290594-595	[53]
	MK790057-059	[56]
	MK560877	[40]
	AB810200-216	[57]

### Phylogenetic analysis

For each genus, a phylogenetic tree was constructed using the Neighbor-joining method and Kimura 2-parameters (K2P) genetic distances with 1000 bootstraps to assess the node robustness with MEGA X software v10.2.4 [58]. A Maximum-Likelihood (ML) tree was also constructed using the same parameters. These tree reconstructions were done with the aim of comparing our sequences to previously published sequences from the vent gastropods of the West Pacific to infer phylogenetic relationships between lineages (Table 3). To do so, the sequences sometimes required to be cut to match the published sequences. Our sequences were submitted to the GenBank Basic Local Alignment Search Tool (BLAST, https://blast.ncbi.nlm.nih.gov) to confirm the morphological identification of each individual. Due to the lack of *Cox1* reference sequences in Genbank for *Provanna*, the taxonomic identification of some of our specimens was limited to the genus level.

To infer the most parsimonious links between mitochondrial haplotypes of different geographic locations within each species or genus, haplotype networks were constructed from our sequences using the Minimum Spanning Network method with the PopART software [59] (http://popart.otago.ac.nz, v4.8.4). Mean genetic distances were calculated between lineages previously identified with NJ and ML trees and haplotype networks using the K2P model of substitutions with 1000 bootstraps using MEGA X software. The Automatic Barcode Gap Discovery method (ABGD), a method used for species delimitation [60], was used with the same model of substitutions and default settings to assess the occurrence of potential cryptic species within each taxa.

Haplotype (*Hd*) and nucleotide (*π*) diversities of each lineage were inferred using DNAsp v5 [61]. To test the hypothesis of demographic changes, the null hypothesis of the mutation-drift equilibrium was tested using Tajima’s *D* and the Fu and Li’s *F* statistics [62]. Distributions of pairwise differences between sequences were established for lineages containing more than 10 sequences, and the observed size-frequency histograms were compared using a chi-square goodness of fit test on counts to the distributions of differences expected under either a constant population size model or an expansion model [63]. Populations satisfying both neutrality tests and a significant fit to the expansion model were considered as presently expanding and the expansion rate was estimated by the statistic Tau *τ*. This statistic can be linked to the time since population expansion using the formula: *τ* = 2μT [64]. In addition, global and/or pairwise *F*_*ST*_ values were computed from haplotype frequencies estimated within mitochondrial clades (i.e. cryptic species) or geographic groups (i.e. metapopulations) when possible and tested against zero (no differentiation) with 1000 permutations of haplotypes between groups using DNAsp v5 [61]. *D*_*XY*_ distances and *Da* net nucleotide divergences between phylogenetic clades were also estimated with the same software.

## Results

A total of 340 *Cox1* sequences were obtained from individuals belonging to the four gastropods families and six genera with fragment lengths varying from 469 to 636 base-pair (bp) (Table 4). Species with dense populations such as *Lepetodrilus schrolli* or *Shinkailepas tollmani* were sub-sampled from the whole collection of sampled individuals. Lineages highlighted by the trees produced by the NJ and ML methods being the same, only NJ trees are shown in the results. ML trees are available in supplementary material (see S1 Fig).

**Table 4:** Variation among *Cox1* nucleotides sequences for each taxon and lineages.

Lineages	Ind.	Seq. (bp)	*H*	*h*	*S*	*s*	*Hd* (± sd)	*π* (± sd)
***Lepetodrilus schrolli complex***	**107**	**620**	**65**	**52**	**66**	**29**	**0.955 ± 0.014**	**0,0162 ± 0,0005**
*L*. *schrolli*	45		31	22	38	24	0.977 ± 0.011	0.0071 ± 0.0004
*L*. aff. *schrolli*	62		34	30	42	27	0.877 ± 0.038	0.0052 ± 0.0007
***Symmetromphalus***	**33**	**629**	**20**	**15**	**58**	**5**	**0.947 ± 0.023**	**0.0353 ± 0.0027**
*S*. *hageni* Manus	9		7	6	7	7		
*S*. aff. *hageni* Woodlark	8		3	2	2	2		
*S*. aff. *hageni* Lau/NFiji	16		10	7	9	6	0.917 ± 0.049	0.0030 ± 0.0004
***Lamellomphalus manusensis***	**29**	**629**	**5**	**3**	**8**	**4**	**0.416 ± 0.107**	**0.0023 ± 0.0007**
*L*. *manusensis* Manus	24		3	2	2	2		
*L*. *manusensis* Futuna	5		2	1	2	2		
***Shinkailepas tufari* complex**	**40**	**636**	**25**	**19**	**119**	**11**	**0.942 ± 0.027**	**0.0581 ± 0.0048**
*S*. *tufari*	19		9	6	11	7	0.778 ± 0.096	0.0024 ± 0.0006
*Shinkailepas sp*.	2		2	2	4	4		
*S*. aff. *tufari* Woodlark	9		6	4	5	4		
*S*. aff. *tufari* Lau/Futuna	3		1	0	0	0		
*S*. aff. *tufari* Lau	7		7	7	16	11		
***Shinkailepas tollmanni***	**75**	**636**	**49**	**42**	**57**	**42**	**0.961 ± 0.014**	**0.0052 ± 0.0004**
***Desbruyeresia***	**24**	**469**	**20**	**17**	**85**	**7**	**0.982 ± 0.018**	**0.0757 ± 0.0072**
*Desbruyeresia melanioides*	14		12	11	22	14	0.967 ± 0.044	0.0098 ± 0.0015
*Desbruyeresia cancellata*	5		4	3	7	6		
*Desbruyeresia costata*	5		4	3	5	4		
***Provanna***	**25**	**530**	**19**	**15**	**55**	**3**	**0.973 ± 0.019**	**0.0484 ± 0.0032**
*Provanna clathrata*	10		9	8	11	7	0.978 ± 0.054	0.0057 ± 0.0011
*Provanna* sp. Woodlark	8		5	3	5	4		
*Provanna* sp. Lau	7		5	4	4	4		

**Ind**: number of individuals; ***H***: number of haplotypes; ***h***: number of unique haplotypes; ***S***: number of segregating sites; ***s***: number of singleton sites; ***Hd***: haplotype diversity; ***π***: nucleotide diversity

### Lepetodrilidae

**Fig 2:**
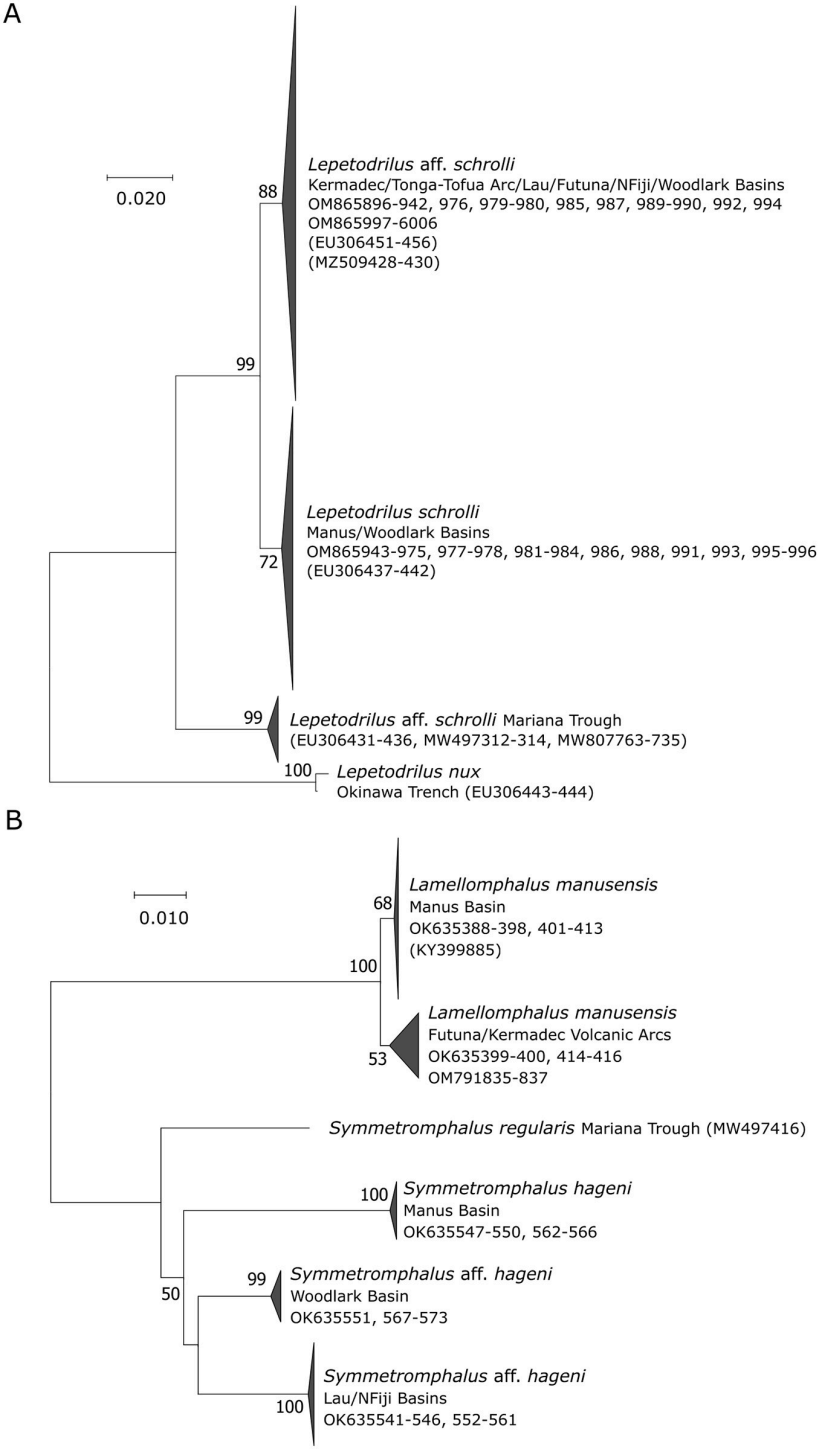
*Cox1* Neighbor-Joining trees inferred from *Cox1* sequences from *Lepetodrilus* (A) and *Symmetromphalus* and *Lamellomphalus* (B) within their family. Number at nodes indicates the proportion of occurrences in 1000 bootstraps; percentages below 50% are not shown. Genbank accession numbers of the present study and published sequences are indicated. Published sequences are in brackets. Sequence lengths: (A) = 496 bp, (B) = 541 bp.

The phylogenetic tree based on *Lepetodrilus Cox1* sequences (Fig 2A) confirmed the occurrence of two strongly supported distinct lineages in the South West Pacific: one with all our samples from the Lau Basin, the Futuna Arc and the North Fiji Basin clustering with *L*. aff. *schrolli* sequences previously obtained by Johnson et al. [17] from the same basins, and another one with all our samples from the Manus Basin, along with *L*. *schrolli* sequences obtained by the same authors from the Manus Basin. Interestingly, sequences of individuals from the Woodlark Basin were sub-divided into these two distinct lineages, indicating that both lineages are sympatric at this site. As observed in Johnson et al. [17], sequences identified as *L*. aff. *schrolli* from the Mariana Trough formed a third lineage which was much more divergent than the two others. These different lineages are clearly different from the other species of *Lepetodrilus* reported in the northwestern Pacific, *L*. *nux*.

**Fig 3:**
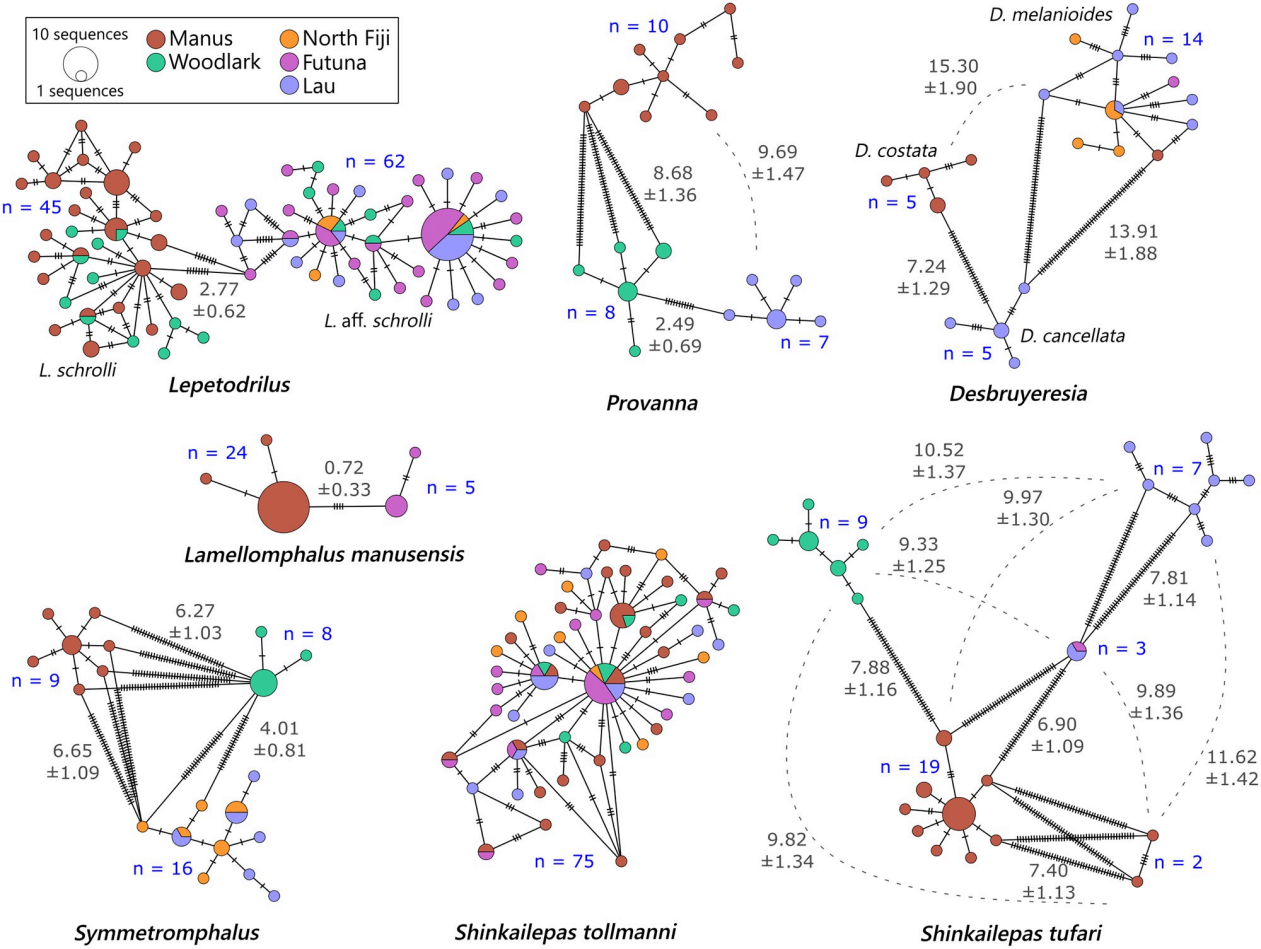
Minimum Spanning Networks based of *Cox1* haplotypes from each taxon. Circles represent the different haplotypes; their diameter indicates the number of individuals with the same haplotype and colour their location. Mutational steps are symbolised by dashes. n = number of sequences within each lineage. Grey numbers represent the divergence between the different lineages (mean K2P% ± Sd).

**Fig 4:**
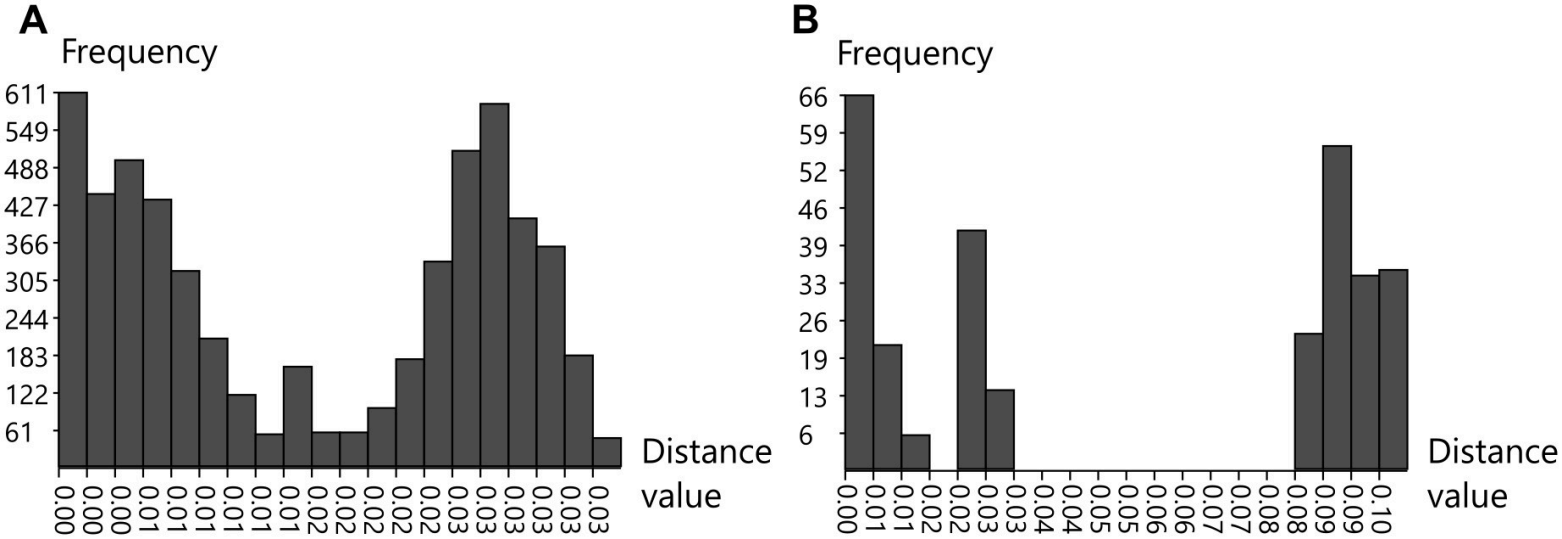
Distribution of pairwise differences obtained from ABGD analyses of *L*. *schrolli* complex (A) and *Provanna* (B) *Cox1* sequences.

Sequences analysis of the 107 specimens from the *Lepetodrilus schrolli* complex of cryptic species revealed 66 segregating sites, which included 37 parsimony-informative sites and 29 singletons across the 620 bp fragment. All mutations were synonymous. The haplotype network clearly separated lineages between the Manus Basin and the eastern basins (i.e. Lau Basin, Futuna Arc and North Fiji Basin), with the Woodlark Basin individuals belonging to both lineages (Fig 3). The two lineages corresponding to two geographic metapopulations were strongly and highly significantly differentiated with *F*_*ST*_ and *D*_*XY*_ equal to 0.772 and 0.027, respectively. At least 4 haplotypes sampled at the Futuna Arc and the Lau Basin displaying a central position in the haplotype network provided an intermediate but specific signature (i.e. a few diagnostic mutations) between the two lineages. Due to these four later individuals, the ABGD analysis did refute that these two lineages correspond to two distinct species (Prior maximal distance *P* = 7.74 10^−3^; Barcode gap distance = 0.024) but the distribution of pairwise differences clearly showed two distinct Gaussian distributions with a small overlap between the intra- and interlineage divergence levels (Fig 4A).

**Fig 5:**
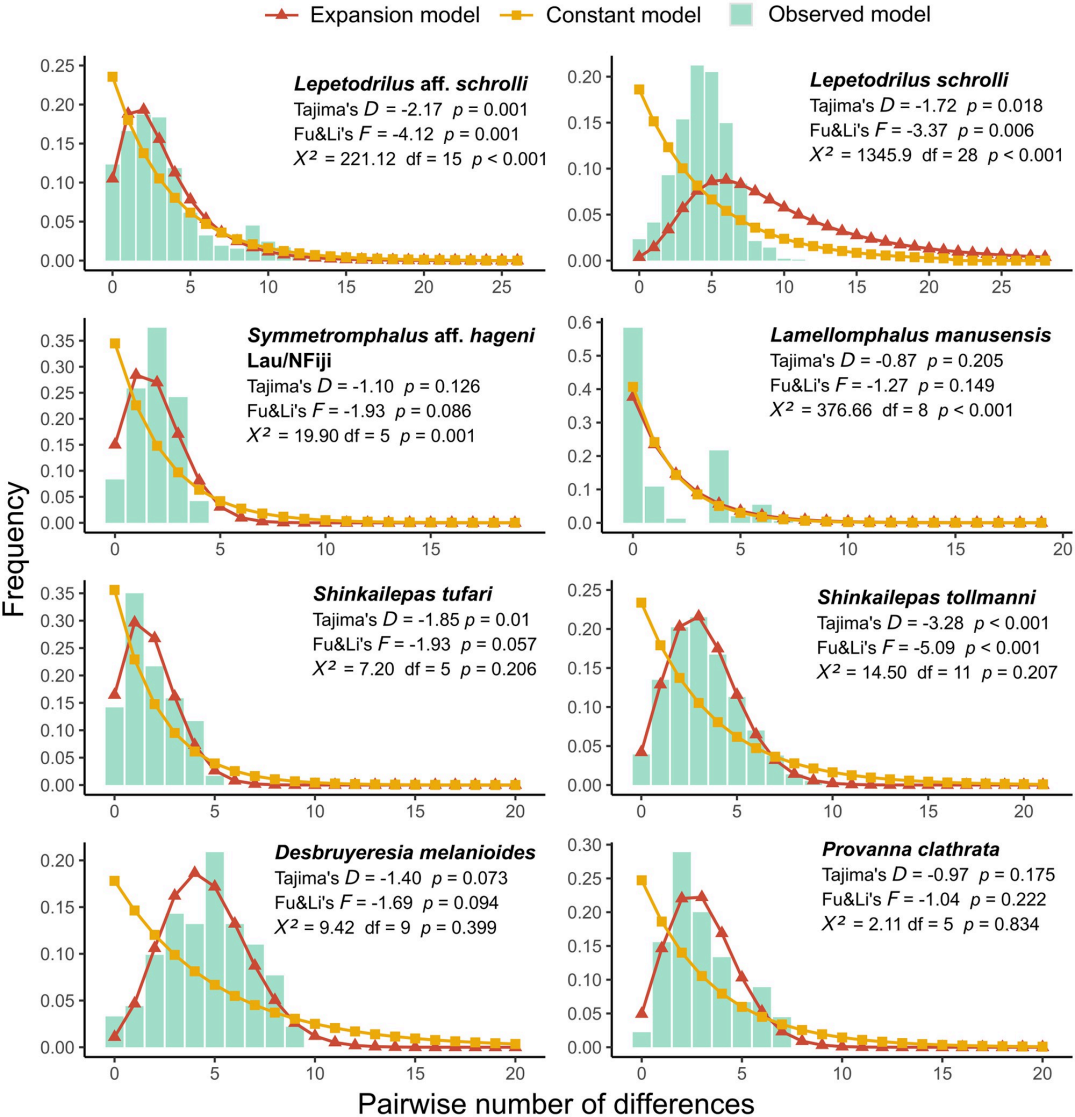
Observed and simulated match-mismatch curves. The chi-square goodness of fit test results presented compare the observed distribution to the expected under an expanding population one. Results of chi-squared test comparing the observed distribution to the expected under a constant population one (not shown in the figure) always indicated that observed distribution did not fit the expected one (*p* < 0.001).

The lineage *L*. aff. *schrolli* haplotype network exhibited a star-like shape with two main haplotypes and numerous derived haplotypes that were shared between the eastern basins populations, but also included some individuals of the Woodlark Basin site. Within this group, one of the two dominant haplotypes was more frequent in the North Fiji Basin and can possibly indicate a slight differentiation between this basin and the Lau Basin/Futuna Arc populations (*F*_*ST*_ = 0.06, *p* > 0.05). The mismatch distribution of *L*. aff. *schrolli* displayed a unimodal shape with an excess of rare variants that fitted well with the model of population expansion if the four intermediate haplotypes are removed (Fig 5). The best fit was obtained with *τ* values ranged between 1.8 and 2, suggesting that the population is expanding since 725 kyr with a mutation rate of 0.2% per site and million years (using a mean rate inferred from Johnson et al. [13, 65] and Breusing et al. [66] with the formula r = D/2T, where D is the species divergence and T the time elapsed since their splitting) if we assume a one-year duration per generation. In the *L*. *schrolli* lineage, the haplotype network was more diversified with much more equally frequent haplotypes shared between the Manus and Woodlark Basins. However, the two basins were significantly differentiated one to each other with a significant *F*_*ST*_ value equal to 0.07 (*p* < 0.05). The mismatch distribution of *L*. *schrolli* displayed a unimodal shape with an excess of rare variants that did not fit either the expected curve under the population expansion model or that of the constant size model (Fig 5). Although significant, Tajima’s *D* and Fu & Li’s *F* statistics were not negative enough to support a recent population expansion with a peak of sequence differences rather centered around 5 mutations (lack of rare/recent mutations in the population but excess of 3–5 bp divergent haplotypes), which could instead indicate that if bottlenecked, this event was too much recent to detect any expansion.

### Neomphalidae

The NJ tree based on *Symmetromphalus* and *Lamellomphalus Cox1* sequences is presented in Fig 2B. This tree includes the only previously published *Cox1* sequence for *Symmetromphalus regularis* from the Mariana Trough and, clearly distinguished this latter species from three strongly supported and divergent lineages in the South West Pacific which were specific to the Manus Basin, the Woodlark Basin, and the eastern basins (i.e. North Fiji and Lau Basins), respectively. The lack of *Cox1* sequences in the Genbank database for the other *Symmetromphalus* species described so far did not allow the identification of the three lineages to the species level. However, given that *Symmetromphalus hageni* was originally described from specimens from the Manus Basin [38], we hereafter use the species name *S*. *hageni* for our Manus Basin samples, and *S*. aff. *hageni* for the two other lineages from the North Fiji-Lau and Woodlark Basins.

The 33 sequences (629 bp long) displayed 58 segregating sites including 53 parsimony-informative sites mostly involved in the divergence of lineages and 5 singletons. Among the 20 haplotypes identified, 15 were unique. Haplotype networks highlighted the co-occurrence of the three lineages identified in the phylogenetic tree, with one or two dominant haplotypes per lineage and a crown of derived haplotypes (Fig 3). The average divergence between lineages within the genus ranged from 4.01 to 6.65%. The net divergence, *Da*, was greater between lineages from the two geographically closer basins of Manus and Woodlark (i.e. 0.52) than between lineages from the Woodlark Basin and the more distant eastern basins (Woodlark Basin vs Lau Basin; *Da* = 0.37). These results were corroborated by the ABGD analysis that showed evidence for three potential cryptic species within our *Symmetromphalus* sequences (*P* = 2.15 10^−2^; Barcode gap distance = 0.021). The overall differentiation between lineages was high with a nearly fixed *F*_*ST*_ value of 0.88. The mismatch distribution was only drawn for *S*. aff. *hageni* from the eastern basins (North Fiji-Lau Basins), for which we had enough individuals. This distribution exhibited a unimodal shape that better fitted the expected curve under the population expansion model (Fig 5). Tajima’s *D* and Fu & Li’s *F* statistics were not significantly different from zero expected from the drift/mutation equilibrium but this might be due to the small sample size we had.

*Lamellomphalus manusensis* also displayed two geographic lineages, one with individuals from the Manus Basin and one with those collected at the Futuna and Kermadec Volcanic Arcs, but these two lineages are much less divergent (i.e. *Da* = 0.0064) than those reported for *Symmetromphalus*. The 29 sequences (629 bp long) displayed as a whole 8 segregating sites including 4 parsimony-informative sites and 4 singletons combined in 5 distinct haplotypes. Because this genus was rather rare, it was not possible to estimate accurately the level of population differentiation associated with the different basins but the divergence between the Manus Basin and the Futuna Volcanic Arc was weak and absent between individuals from the Futuna and Kermadec Volcanic Arcs.

### Phenacolepadidae

**Fig 6:**
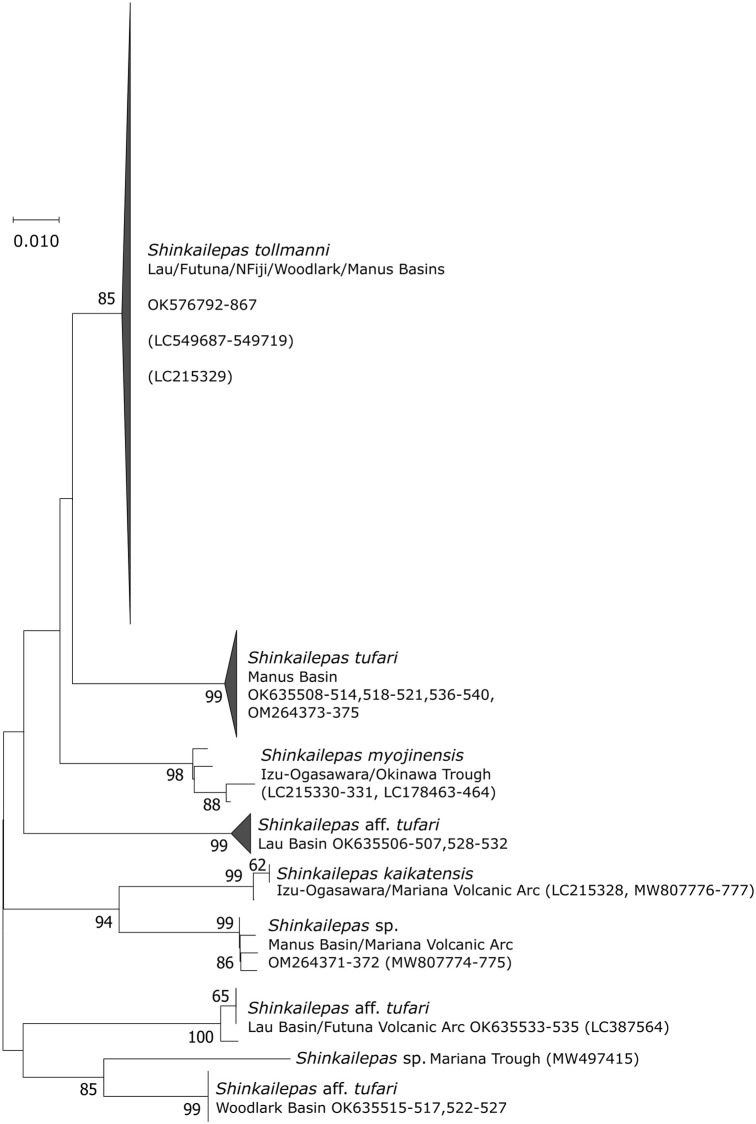
*Cox1* Neighbor-Joining trees showing the position of individuals from *Shinkailepas* within their genus. Numbers at nodes indicate the proportion of occurrences in 1000 bootstraps; percentages below 50% are not shown. Genbank accession numbers of the present study and published sequences are indicated. Published sequences are in brackets. Sequence lengths: 281 bp.

Within the genus *Shinkailepas*, the NJ tree clearly distinguished nine lineages involving four valid and well-known species in the West Pacific: *S*. *tollmanni*, *S*. *myojinensis*, *S*. *kaikatensis* and *S*. *tufari* and two undescribed species from both the Mariana Trough and the Mariana Volcanic Arc (Fig 6). Among individuals morphologically identified as *S*. *tufari* from the CHUBACARC cruise, four lineages clearly discriminated from the North West Pacific species. The species name *S*. *tufari* is hereafter used for the Manus Basin specimens as this basin corresponds to the type locality of the species [37]. Other putative lineages of *S*. *tufari* found in the other basins have been then referenced as *S*. aff. *tufari*. One distinct lineage, with only two individuals, was however also found in the Manus Basin. This rare lineage first identified as *S*. *tufari* based on morphological characteristics merged with *Shinkailepas sp*. individuals from the Mariana Volcanic Arc and should represent another geographic species. This lineage is related to *S*. *kaikatensis* reported in the Izu-Bonin-Mariana Volcanic Arc.

Among the 40 specimens identified as *S*. *tufari sensu lato*, 119 segregating sites were reported including 108 parsimony-informative sites mostly involved in the lineage divergence. All but one mutation were synonymous, and the non-synonymous one is unique to the two individuals from *Shinkailepas sp*. lineage (Table 4). Out of these *tufari*-like sequences, the lineages were sub-divided into 25 haplotypes among which 19 were unique. The haplotype network highlighted the distinct geographical distribution of the five lineages with average divergences ranging from 6.9% to 11.62% (Fig 3). These lineages were also supported by the ABGD analysis (*P* = 5.99 10^−2^; Barcode gap distance = 0.041) confirming the existence of 5 putative cryptic species. Three lineages were only reported from one basin (Manus (defined as *S*. *tufari*), Woodlark and Lau Basins: see Table 4) but the lineage typical of the Futuna Volcanic Arc was also collected in the Lau Basin. In contrast to *Symmetromphalus*, divergence was greater between the Lau and Woodlark Basins (net divergence *Da* = 0.090) than between the Manus and Woodlark Basins (*Da* = 0.071) with the near fixation of haplotypes between basins (*F*_*ST*_ = 0.95; *p* < 0.001). The mismatch distribution of *S*. *tufari* best fitted the expected curve of population expansion, despite non-significant Tajima’s *D* and Fu & Li ‘s *F* statistics. The non-deviation of the two tests from neutral expectations may therefore be attributable to the small number of sequences available (Fig 5).

In contrast to *S*. *tufari* complex, both the NJ tree and the haplotype network clearly indicated that *S*. *tollmanni* specimens from all basins and the Futuna Volcanic Arc belong to a single phylogenetic lineage which also comprises the *S*. *tollmanni* sequences from Yahagi et al. [29] and Fukumori et al. [50] (Figs 3 and 6). The analysis of the 75 *S*. *tollmanni* sequences led to 57 segregating sites including 15 parsimony-informative sites and 42 singletons forming 49 distinct haplotypes. All but one mutation were synonymous, and the non-synonymous one was a singleton. No genetic differentiation was found between the four basins and the Futuna Volcanic Arc (*F*_*ST*_ = -0.0037; *p* > 0.05). The haplotype network had a star-like shape with the most dominant haplotype being shared between all sampled localities. The occurrence of a single lineage was also supported by the small genetic divergence between individuals (i.e. 0.52%, Table 4). The mismatch distribution was unimodal with an excess of rare variants that fitted the expected curve of population expansion (Fig 5). This was also supported by significant negatives Tajima’s *D* and Fu & Li’s *F* statistics. The best fit was obtained with a *τ* value of 2.8, suggesting that the population is expanding since 1.1 My with a mutation rate of 0.2% per site and million years if we assume a one-year duration per generation.

### Provannidae

**Fig 7:**
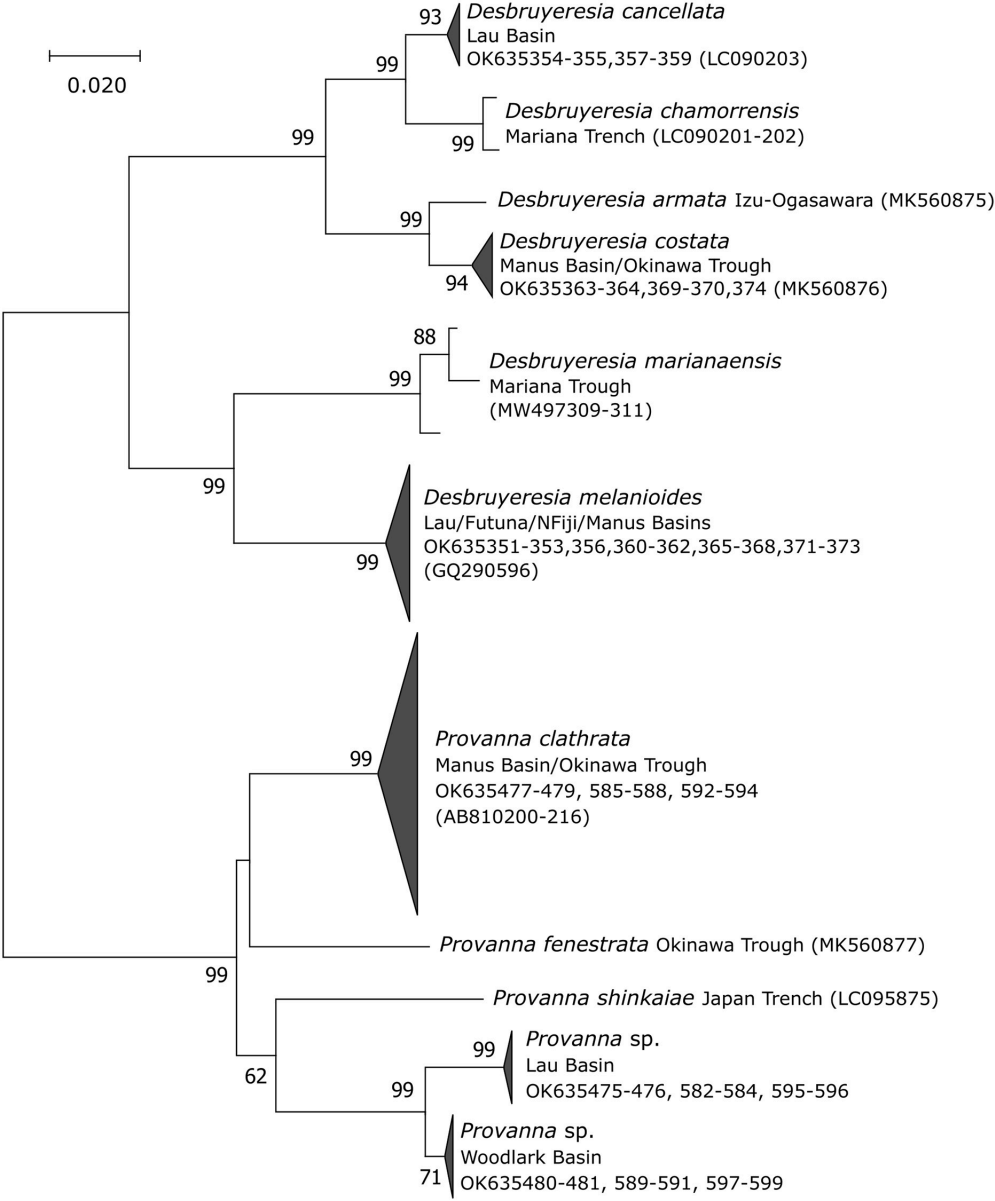
*Cox1* Neighbor-Joining trees showing the position of individuals from *Desbruyeresia* and *Provanna* within their family. Number at nodes indicates the proportion of occurrences in 1000 bootstraps; percentages below 50% are not shown. Genbank accession numbers of the present study and published sequences are indicated. Published sequences are in brackets. Sequence lengths: 469 bp.

The NJ tree based on *Desbruyeresia* and *Provanna Cox1* sequences is presented in Fig 7. Within the genus *Desbruyeresia*, the phylogenetic tree exhibited 6 main lineages corresponding to 6 morphologically described species. Three are exclusive to the North Pacific (i.e. *D*. *marianaensis*, *D*. *armata*, and *D*. *chamorrensis*) and two are from the South Pacific (i.e. *D*. *melanioides* and *D*. *cancellata*) (Fig 7). The last species (i.e. *D*. *costata*) was reported in both the North and the South Pacific. Our western Pacific samples fell within 3 of these morphological species (i.e. *D*. *melanioides*, *D*. *cancellata* and *D*. *costata)*.

The analysis of the 24 *Desbruyeresia* sequences collected during the CHUBACARC cruise diplayed 85 segregating sites including 78 parsimony-informative sites, which were nearly all involved in species divergence. All mutations were synonymous. Seventeen of the 20 haplotypes were unique. The haplotype network was performed on the three main lineages, which corresponded to *D*. *melanioides*, *D*. *cancellata* and *D*. *costata* (Figs 3 and 7). The average divergence between these 3 species ranged from 7.2 to 15.3% with nearly fixed haplotypes (*F*_*ST*_ = 0.94; *p* < 0.001). *Desbruyeresia costata*, a species originally described from the Okinawa Trough [40], exclusively occurred in the Manus Basin, while *D*. *cancellata* was sampled in the Lau Basin. Only one individual of *D*. *cancellata* was morphologically identified from the North Fiji Basin but was not barcoded. Conversely, the third species *D*. *melanioides* was widely distributed across the eastern localities (i.e. Lau Basin, Futuna Volcanic Arc and North Fiji Basin) and displayed no spatial genetic structure in the sampled area. The number of haplotypes was however too small to produce accurate *F*_*ST*_ estimates between basins for this later species. Based on morphology, we also identified one individual from *D*. *melanioides* at the Woodlark Basin site, but it was not barcoded. The mismatch distribution was unimodal with an excess of rare variants and fitted the expected curve of population expansion (Fig 5). Tajima’s *D* and Fu & Li’s *F* statistics, although negative, were not significant probably because of the small number of sequences available.

Genetic analyses of specimens from the genus *Provanna* also indicated species diversification at the scale of the West Pacific. Three distinct lineages can be depicted from the NJ tree, one being closer to the previously described species in the Okinawa Trough, i.e. *P*. *fenestrata*. This lineage corresponds to the species *Provanna clathrata* described from the Okinawa Trough. The two others unnamed lineages were closer to *P*. *shinkaiae* also found in the northwestern Pacific. The lack of *Cox1* sequences in the Genbank database for the western Pacific species did not allow to identify our specimens to the species level within this genus. As already reported for *Desbruyeresia*, this genus comprises one lineage composed of individuals from the Manus Basin and the Okinawa Trough. The 25 *Provanna* individuals sampled during the CHUBACARC cruise exhibited 55 segregating sites, which included 52 parsimony-informative sites mostly involved in the lineage divergence. Only one mutation was nonsynonymous and shared by all specimens coming from the Manus Basin. Over the 19 haplotypes identified, 13 were unique. The haplotype network identified three geographic lineages endemic to the Manus, Woodlark and Lau Basins respectively (Fig 3). The average divergence between these lineages ranged from 2.49 to 9.69% with more divergence accumulated between the Manus and Woodlark Basins (*Da* = 0.077) than between the Lau and Woodlark Basins (*Da* = 0.022). This geographic pattern of divergence was quite similar to *Symmetromphalus* (see: Table 4) with again nearly fixed haplotypes between basins (*F*_*ST*_ = 0.95; *p* < 0.001). The ABGD analysis suggested the co-occurrence of two potential cryptic species, with individuals segregating between the Manus Basin and the Woodlark/Lau Basins (*P* = 2.15 10^−2^; Barcode gap distance = 0.054) by a clear barcode gap in genetic distances of 0.04 and 0.08 (Fig 4B). The mismatch distribution obtained from the most sampled species (i.e. *Provanna clathrata* collected at the Manus Basin) exhibited an excess of rare variants and better fitted the expected curve of population expansion (Fig 5). Tajima’s *D* and Fu & Li’s *F* statistics were however not significant, likely due to the small sample size.

## Discussion

Assessing the geographical range of species is essential in understanding how communities are structured and how they should eventually be protected. Cryptic species, highlighted through barcoding approaches, are common at deep-sea hydrothermal vents [14, 17–20]. When left undetected, their occurrence can lead to an underestimation of biodiversity or incorrect species distribution boundaries. In this study, *Cox1* barcoding analyses provided new insights into the distribution range of several gastropod species across the South West Pacific. While the concept of species has been strongly debated for decades [67, 68], following the ABGD approach/definition for species delimitation, cryptic species were found within two taxa, *Shinkailepas tufari* and *Symmetromphalus hageni*. These two complexes included species restricted to the Manus Basin, the Woodlark Basin, and one or several of the eastern basins including the Lau Basin and the North Fiji Basin, and the Futuna Volcanic Arc. These findings are in agreement with the notion of geographic species and the concept of the stepwise colonisation of deep-sea vents, at least in the discontinuous systems of the western Pacific. *Symmetromphalus regularis*, described from the Mariana Trough, has been reported at the Lau and North Fiji Basins [16], however, individuals from this study are clearly distinct from this species, suggesting that *S*. *regularis* is absent from the South West Pacific. Further morphological observation might help to identify the different species, although shell plasticity seems to occur, especially in *Symmetromphalus* where shell shape and thickness were highly variable among Manus Basin individuals (C. Poitrimol, pers. obs.).

The genus *Provanna* depicted a similar pattern with three distinct lineages although the ABGD method only detected two potential species, individuals from the Lau and Woodlark Basins belonging to the same species, yet with a complete geographic isolation of these populations. Indeed, the intraspecific divergence represented in the pairwise distance histograms, ranging from 0 to 3% of divergence, was composed of two modes separating individuals from the two basins. The average divergence between these two lineages (i.e. 2.5%) is in the same order of magnitude as the one segregating *Symmetromphalus* species between the same basins (i.e. 4%), suggesting that they could share the same evolutionary history of populations. The lack of significance of the barcode gap method between these two geographic populations of *Provanna* may be due to the very small number of individuals analysed, leading to an underestimation of the haplotype diversity. A higher sampling effort will increase the statistical power of the AGBD with a more accurate information on the status of the two lineages. The two unnamed *Provanna* species/subspecies are probably attributable to the *Provanna* species already described from the western Pacific region: *P*. *segonzaci* in the Lau Basin and *P*. *buccinoides* in the Lau and North Fiji Basins but also *Provanna nassariaeformis*, originally described from the Mariana Trough and further reported in the Manus Basin [16]. The lack of sequences in Genbank for these latter species prevented us from identifying our specimens with the original descriptions. Although we did observe morphological (shell) differences between our samples, original descriptions were based on small individuals and do not describe morphological ontogenic changes. Diagnostic characteristics in *Provanna* species [42, 69] notably involve the number of spires and ornaments on the shell, but our barcoding results clearly showed that this number changes as the animal grows and might thus not represent a reliable trait for morphological identification (C. Poitrimol, pers. obs.). Other genes need to be investigated to confirm species delimitations. Also, further morphological observation of the shell microstructures, soft body parts or radula could reveal some variations between the putative species and refine their initial descriptions. For example, while *Alviniconcha* species have long been considered cryptic, closer examination revealed that they are morphologically distinguishable [33, 70].

In contrast to the geographic species complexes depicted in morphological species such as *S*. *tufari* or *S*. *hageni*, other gastropod species indicated a higher level of connectivity between some basins. *Lepetodrilus schrolli* has previously been identified as cryptic, with *L*. *schrolli* occurring at the Manus Basin and *L*. aff. *schrolli* in the Lau and North Fiji Basins [17], but *L*. aff. *schrolli* was then also identified at the Manus Basin [19]. Our results confirmed the existence of two lineages separating the Manus Basin from the North Fiji and Lau Basins and the Futuna and Kermadec Volcanic Arcs (Fig 2A). The two lineages and/or subspecies are however mixed at the newly discovered site at the Woodlark Basin (present study). This can reinforce previous barcoding analyses of Plouviez et al. [19] who did find *L*. aff. *schrolli* at the opening of the Manus Basin on the flanks of the Susu volcanoes. According to our ABGD analysis, the two lineages may still belong to a single species that experienced spatial isolation of its populations with possible secondary contacts, at least in the Woodlark Basin. Secondary contacts with admixtures might be probable with the superimposition of lineages at some locations, and could explain why we have found putative intermediate haplotypes between *L*. *schrolli* and *L*. *aff*. *schrolli* at the Futuna Volcanic Arc sites. This has been previously shown between overlapping lineages of *Lepetodrilus elevates* at 9°50N on the East Pacific Rise [20]. Further genetic studies involving a greater number of nuclear markers are however needed to clarify the status of this species. Interestingly, the level of divergence between the two lineages is similar or close to those observed between the *Provanna* eastern species and the Woodlark and Lau/Fiji Basins species of *Symmetromphalus*. This observation could reflect similar evolutionary histories of vicariance between different taxa associated with an ongoing speciation of these taxa in allopatry and the central role of the Woodlark Basin in allowing both the dissemination and the faunal connection of different basins.

While various distribution patterns emerged among gastropod taxa, phylogeographic breaks consistently occur between the Manus Basin and/or the Woodlark Basin and the other eastern populations from South West Pacific (i.e. *Symmetromphalus*, *Provanna*, *Lepetodrilus* and *Shinkailepas* species complexes). In addition, levels of divergence were not correlated with geographic distances. A geographic split between populations of the Manus Basin and the North Fiji/Lau Basins was already reported for a number of taxa, including *Ifremeria nautilei* (a large symbiotic gastropod), the shrimp *Rimicaris variabilis*, the crab *Austinograea alayseae* and the limpet *Lepetodrilus schrolli* [19, 30–32, 71]. This study is however the first to publish genetic data associated with the Woodlark Basin and its stepping-stone role on the vent fauna dissemination. Geological events or hydrographic barriers can contribute to explain the vicariant events that caused these geographic isolations. Physical barriers created by the New Guinea archipelago have already been proposed to explain the Manus Basin vent fauna isolation [30]. In addition, a major reorganisation of the plates was initiated in the western Pacific about 25 million years ago (Mya) [23]. The opening of actual southwestern basins is relatively recent (<10 My): the Woodlark Basin, the oldest, opened 5–7 Mya, followed by the Manus Basin (5 Mya), the North Fiji Basin (3–4 Mya) and the Lau Basin (1–2 Mya). These geological rearrangements, although progressive, could have led to a step-by-step ridge colonization and further species isolation by vicariance. The range of divergences observed between cryptic lineages of the different gastropod taxa is probably indicative of a shared evolutionary history at different times of the geological formation of these basins (i.e. 2.5–4%; 6–10%; 14–15%, Table 4). Assuming a mean substitution rate of 0.2% per million years across gastropod species [13, 66] the geographic lineages would have diverged respectively between 6–10, 15–26 and 35–38 Mya. Although divergence times must be considered with caution, as substitution rates can vary depending on taxa, the opening of the South Fiji Ridge and the associated Solomon Basin 25 Mya ago and its further subduction leading to the present-day basins about 10 Mya ago could have played a major role for the two most recent series of divergences found in the vent fauna, which could be more obviously retrieved in the poor-dispersive species. These divergence times between geographic lineages were also concordant with reported diversification events of deep-sea organisms during the Oligocene and Miocene [72, 73].

The pattern and level of population divergence however differed among species, and could be explained by biotic factors inherent to species ecology and life-history traits such as reproduction and larval biology which affect larval dispersal. Contrasted phylogeographic patterns also occurred between closely related species. This pattern was already reported in the genus *Alviniconcha* in this region, with *A*. *kojimai* and *A*. *boucheti* largely distributed along the Manus, North Fiji, and Lau Basins, and the Futuna Volcanic Arc, while *A*. *strummeri* has a more limited distribution to the eastern basins [13, 33, 70]. This pattern is partly surprising as closely related species are expected to share reproductive traits that are often phylogenetically constrained [74–76]. Surprisingly, patterns of differentiation repeatedly differ between congeneric species in several gastropod genera with two alternative and opposed dispersal strategies. While *Shinkailepas tollmanni*, *Lamellomphalus manusensis* or *Desbruyeresia melanioides* are widely distributed across the South West Pacific basins with very weak divergence between geographic populations, other species from the same genera or family were limited to nearly each basin (i.e. *Shinkailepas tufari* and *Symmetromphalus hageni* complexes of species, *Desbruyeresia cancellata*). Such variability in the distribution patterns of closely related species were also reported in other groups of benthic organisms such as the deep-sea stalked barnacles in the West Pacific. For instance, the genera vent neolepadid barnacles *Vulcanolepas*/*Leucolepas* display high species diversity with one species per basin: *V*. *buckeridgei* in the Lau Basin, *V*. *fijensis* in the North Fiji Basin, a new undescribed *Vulcanolepas* species in the Woodlark Basin, *Leucolepas longa* in the Manus Basin, *V*. *oshaeai* in the Kermadec Volcanic Arc, and *V*. *verenae* in the Mariana Trough [35, 77–79]. On the contrary, the non-hydrothermal deep-water stalked barnacle *Scalpellum stearnsii* forms a complex of 4 cryptic species with one species present throughout the South West Pacific, from Papua New Guinea to Fiji [73]. Finally, intermediate patterns were also highlighted on non-hydrothermal bathyal barnacles in the genus *Waikalesma* with two species, i.e. *W*. *boucheti* and *W*. *dianagonesae*, present in sympatry in Papua New Guinea but with different geographic distribution [80].

Currents will differentially affect larval dispersal potential, and hence species range, depending on closed interactions with the planktonic larval duration, larval behaviour and the depth at which they disperse [81]. While species with lecithotrophic larvae, potentially associated with short Pelagic Larval Duration (PLD), would have a limited distribution range, species with planktotrophic larvae, associated with a longer PLD, are expected to be widely distributed if not lost from active areas. However, such strategies may lead to opposite larval duration depending on the depth at which larvae are able to move as the low temperature near the seafloor may allow lecithotrophic larvae to have longer PLDs than planktotrophic larvae because of their reduced metabolism [82, 83]. In addition, some larvae like those of the vent polychaete *Alvinella pompejana* may arrest their development in cold water until suitable conditions are encountered [84, 85], resulting in long-lasting propagules. *Shinkailepas* and *Desbruyeresia* larvae have been shown to be planktotrophic [29, 40, 42, 52] but most of their congeneric species exhibit highly fragmented genetic structure and species diversification. Variable larval lifespan and/or ontogenic behaviour could explain the contrasted distributions within these genera, as connectivity between basins would vary according to PLD and depth of dispersal [28]. The South Equatorial Current may connect all South West basins once every hundred of thousands of years via stepping-stones for species with long larval lifespan (170 days at 1 000 m) [28]. Connection is predicted to be successful westward from the Woodlark Basin to the Manus Basin, and from the Lau to the North Fiji Basins, once every ~5 000 to ~12 000 years considering a pelagic larval duration of 82 days between 100 to 1 500 m [28]. However, eastward gene flow from the Manus Basin to the North Fiji and Lau Basins has been suggested for several taxa [19, 26, 30, 31] but not *Ifremeria nautilei* when looking at more contemporary gene flow [71]. Different larval behaviours have already been observed between North West Pacific *Shinkailepas* species [52, 86]. While for some species larvae are believed to migrate to the surface, thus taking advantage of food resources and stronger currents, others remain close to the seabed, inducing different dispersal capacities. Vertical migration to shallow water has been suggested for *Shinkailepas tollmanni* for which larvae, although encapsulated until they reach their trochophore stage, are thought to stay pelagic for over a year [29], potentially allowing long-distance dispersal through the Manus, Woodlark, North Fiji, and Lau Basins, and the Futuna Volcanic Arc. Conversely, in the specific case of *Shinkailepas tufari*, larvae might be more likely to stay near the seabed which could account for their more limited distribution ranges. *Symmetromphalus*, *Provanna* and *Lepetodrilus* have lecithotrophic larvae [19, 38, 42, 55, 87]. *Lepetodrilus schrolli* is widely distributed from the Manus Basin to the Kermadec Volcanic Arc, a rather wide distribution for a non-feeding larva. The low temperature of deep waters or delayed larval development behaviour could play a central role in the species distribution with some basins acting as stepping-stones [28]. Finally, differences in dispersal might be related to offspring availability, a species with high occupancy and high fecundity being able to produce more larvae over time and space [88]. Species occupancy indeed results from their success in colonising habitat but also on the availability and frequency of their habitat.

Finally, our results raise questions about the faunal links between the two distinct biogeographic provinces of the North West and the South West Pacific [7, 89]. While separation of species was generally greater between the North West and the South West Pacific than within the South West Pacific, the provannid gastropods, *Desbruyeresia costata* and *Provanna clathrata* from the Manus Basin, have a limited distribution range across the South West back-arc basins but display connections with the Okinawa Trough. In the same way, *Shinkailepas* sp. found in the Manus Basin was previously described from the northwestern Pacific. Even with larvae dispersing almost one year at a depth of 100 m, populations from the Manus Basin are not connected to those of the Mariana Trough, where the nearest northern vent sites are found to date [28]. Considering the large geographic gap between the South West and North West regions of the Pacific, the link between the Okinawa Trough and the Mariana Trough with the Manus Basin implies the occurrence of yet undiscovered sites that could act as stepping-stones, throughout the Philippine Arc for instance. In comparison, analysis of the distribution of hydrothermal and non-hydrothermal deep-sea barnacles showed distinct distributions of closely related species between the northern and southern West Pacific [73, 79].

Our results also support the hypothesis introduced by Boulart et al. [35] establishing that the Woodlark Basin could act as a biological stepping-stone. Depending on taxa, populations from the Woodlark Basin are either closer to the ones in the eastern basins (*e*.*g*. *Symmetromphalus* aff. *hageni* and *Provanna sp*.), or closer to those of the Manus Basin (*S*. *tufari*). For other species (e.g. *S*. *tollmanni*, *D*. *melanioides*) the Woodlark Basin appears to be widely connected with all other basins, consisting of a metapopulation in the region as also observed for *Ifremeria nautilei* [35], or exhibit lineages from both the North Fiji/Lau Basins and the Manus Basin (e.g. *Lepetodrilus schrolli* complex). The Woodlark Basin hence seems to act as an intermediate between the Manus Basin and the eastern regions including the North Fiji and Lau Basins or the Futuna Volcanic Arc but also constitutes a contact zone for some taxa.

To conclude, new cryptic species are likely to co-occur over the complex system of disconnected basins typical of the West Pacific. Most of them are geographic and strengthen the hypothesis of speciation in allopatry as a by-product of the plate tectonism. Very contrasted phylogeographic patterns are however observed within hydrothermal vent gastropods from southwestern Pacific back-arc basins and suggest that species may have evolved under contrasted if not opposite dispersal strategies: a situation that could be favoured by fragmentation and habitat instability. Connectivity between basins is therefore highly variable according to species and their early life history traits. So far, larval lifespan and behaviour are poorly known for many vents species and seem to greatly differ even between congeneric species. Understanding the colonisation potential of vent species from their life history traits could help to improve larval dispersal modelling studies and thus better understand connectivity between basins [81]. Our results have an important impact in terms of biological conservation: as species colonisation potential is highly variable, susceptibility to deep-sea mining will be different between taxa, with a great proportion of vulnerable species which seem to poorly disperse. Anticipation of the effect of mining will therefore require the study of all the species of the ecosystem and should integrate species with the lowest dispersal ability. In addition, due to the intermediate position of the Woodlark Basin in connecting the western Pacific basins, mining vent sites in this basin could potentially influence the ‘rescue’ effect of any of the other basins on the Manus Basin vent communities.

## Supporting information

S1 Fig *Cox1* Maximum Likelihood trees inferred from *Cox1* sequences from *Lepetodrilus* (A), *Symmetromphalus* and *Lamellomphalus* (B), *Shinkailepas* (C) and *Desbruyeresia* and *Provanna* (D) within their genus or family. Number at nodes indicates the proportion of occurrences in 1000 bootstraps. Genbank accession numbers of the present study and published sequences are indicated. Published sequences are in brackets. See NJ trees for sequence lengths.
(PDF)
