# Supplementary figures and images for: Contrasted phylogeographic patterns of hydrothermal vent gastropods along South West Pacific: Woodlark Basin, a possible contact zone and/or stepping-stone
Source: PLoS One. 2022 Oct 5;17(10):e0275638. doi: 10.1371/journal.pone.0275638 (PMC9534440; doi:10.1371/journal.pone.0275638)

A

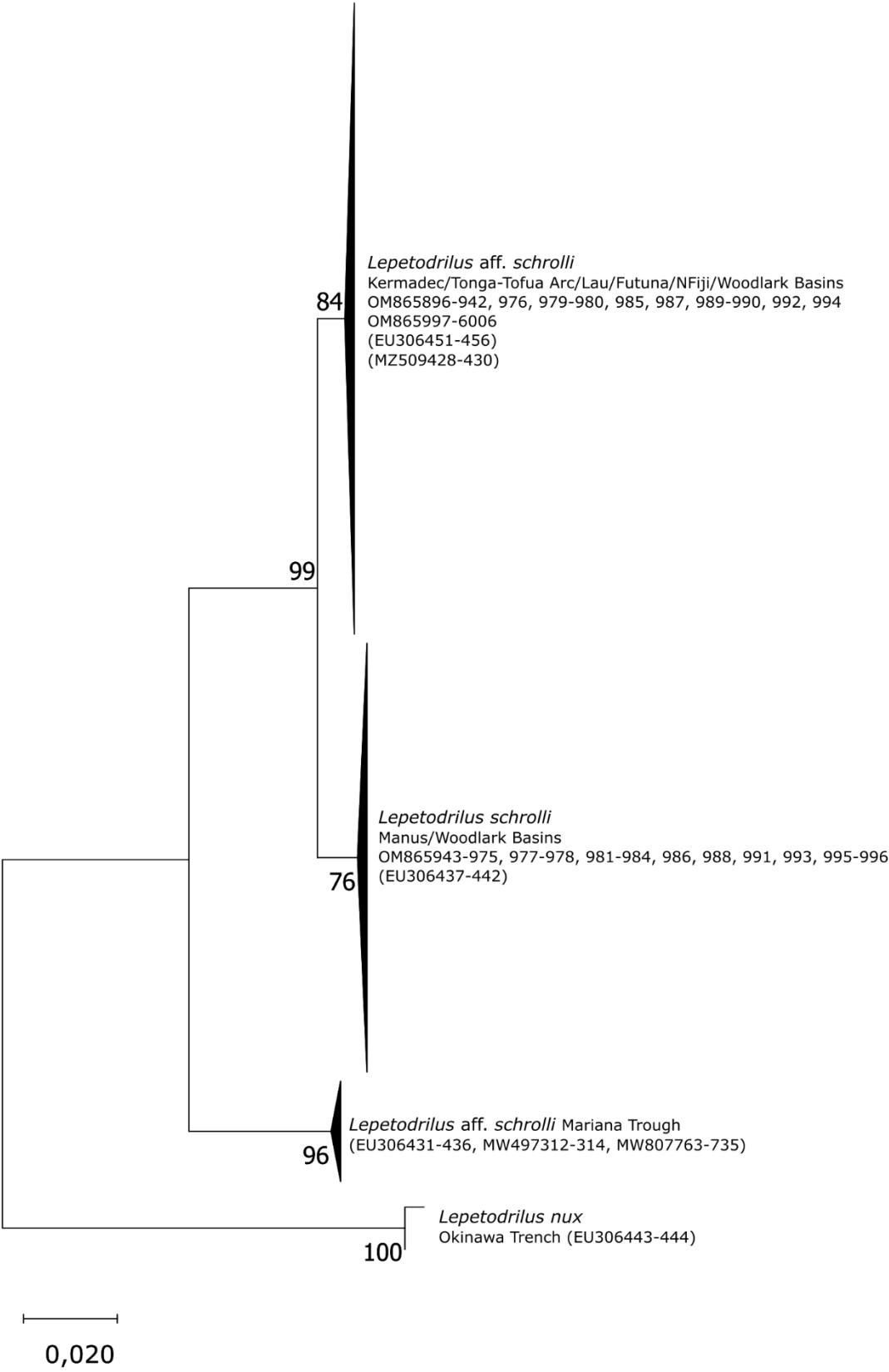

B

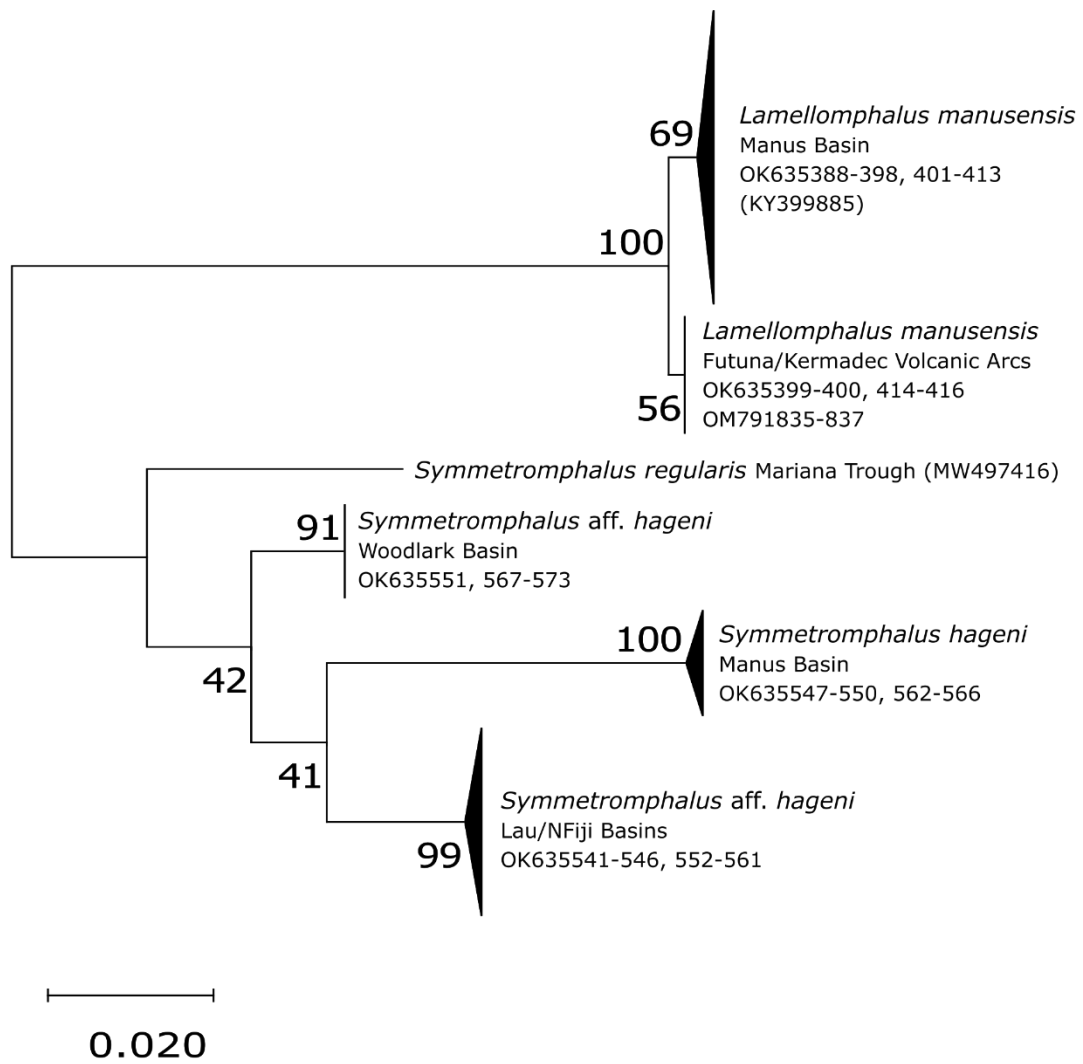

C

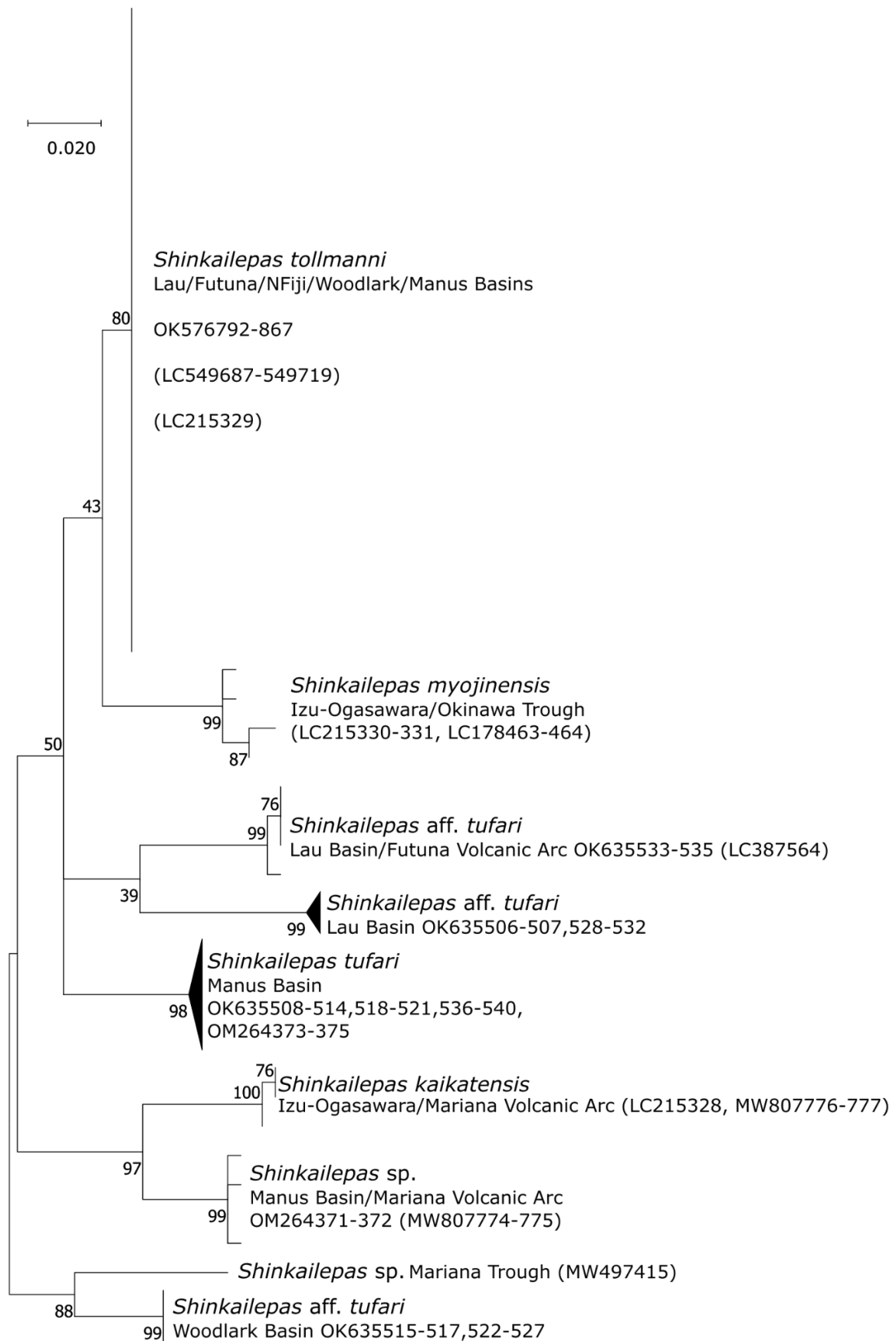

D

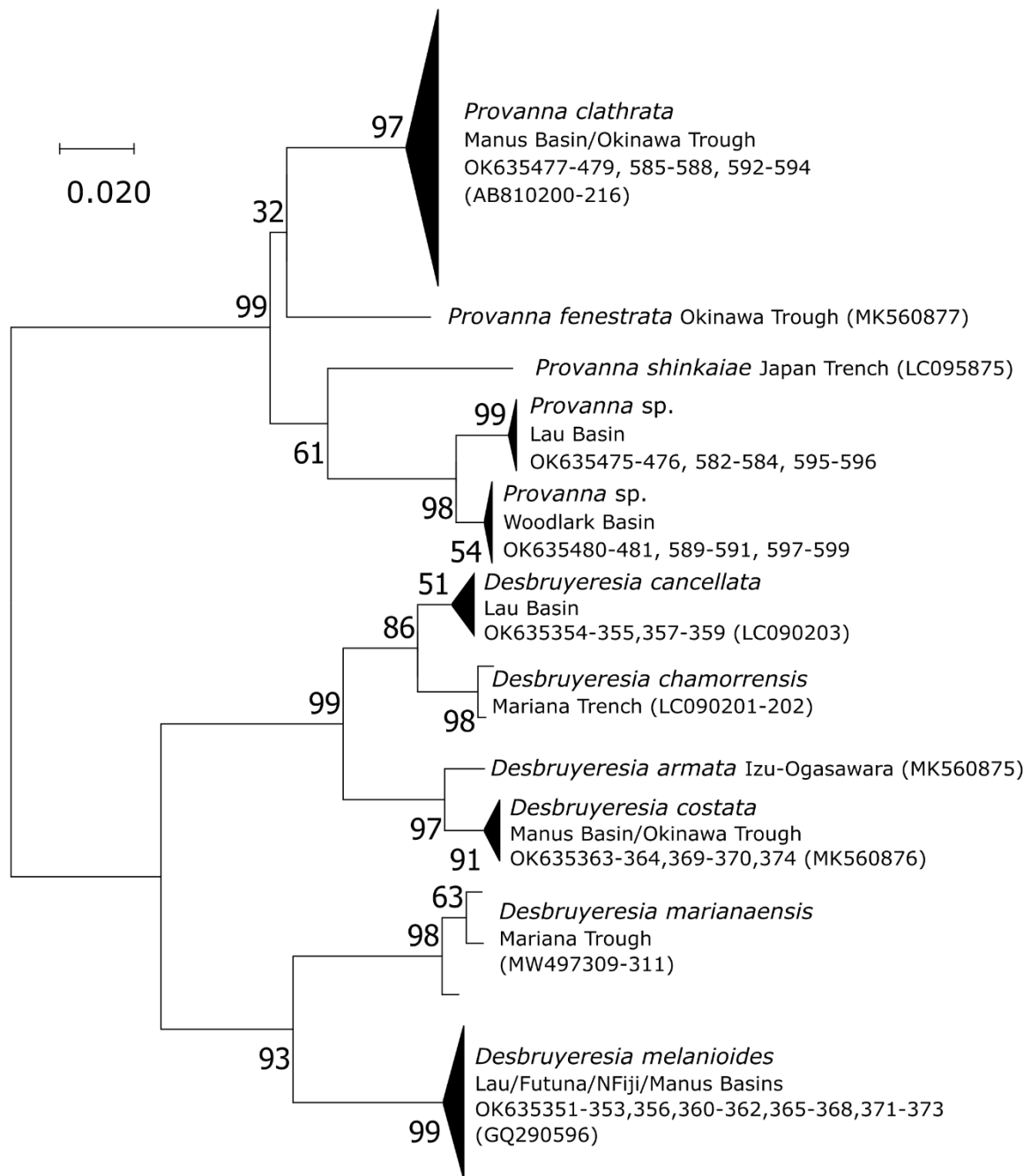

Supplement: S1 Fig — Number at nodes indicates the proportion of occurrences in 1000 bootstraps. Genbank accession numbers of the present study and published sequences are indicated. Published sequences are in brackets. See NJ trees for sequence lengths. (PDF) [file pone.0275638.s001.pdf]
